# Spontaneous L4/L5 Dislocation Associated With Diffuse Idiopathic Skeletal Hyperostosis in a Young Patient With Severe Obesity: A Case Report

**DOI:** 10.7759/cureus.105428

**Published:** 2026-03-18

**Authors:** Ikuyo Tamatani, Atsuyuki Kawabata

**Affiliations:** 1 Orthopaedics, Kudanzaka Hospital, Tokyo, JPN

**Keywords:** cauda equina syndrome (ces), diffuse idiopathic skeletal hyperostosis (dish), lumbar dislocation, neurological deficit, spinal pseudoarthrosis

## Abstract

Diffuse idiopathic skeletal hyperostosis (DISH) is characterized by extensive ossification of the spinal ligaments and entheses, resulting in a rigid spine that behaves biomechanically like a long bone. Although spinal fractures and dislocations after minor trauma are well recognized in ankylosed spines, non-traumatic spontaneous instability is rare. We report a rare case of spontaneous L4/L5 dislocation in a young patient with severe obesity and DISH.

A 40-year-old man presented with progressive low back pain, bilateral lower extremity weakness, and bladder-bowel dysfunction. He had initially been treated at another hospital for septic shock due to urinary tract infection, but his neurological deficits persisted after improvement of the infection. On transfer to our institution, he was 168 cm tall, weighed 132 kg, and had a body mass index of 46.7 kg/m^2^. Neurological examination showed severe bilateral lower extremity weakness and sensory disturbance below the L1 level. Computed tomography demonstrated extensive thoracolumbar ankylosis consistent with DISH, with the L4/L5 segment representing the only remaining mobile level. An L5 superior articular process fracture, facet joint separation, and spontaneous L4/L5 dislocation were identified. Magnetic resonance imaging showed cauda equina compression at L4/L5. Because the lesion was considered highly unstable, staged circumferential reconstruction was performed. Initial percutaneous posterior fixation from L2 to S1 was carried out in the lateral decubitus position to avoid aggravation of the dislocation that might have occurred in the prone position. Pelvic fixation with S2 alar-iliac screws was then added in a second stage after repositioning the patient prone, because screw insertion was technically difficult in the lateral position. Anterior lumbar interbody fusion at L4/L5 with iliac bone grafting was subsequently performed. At six months postoperatively, low back pain had improved, and local stability was maintained, although severe motor deficits and bladder-bowel dysfunction persisted.

In this patient, severe obesity and extensive ankylosis likely concentrated chronic mechanical stress at the L4/L5 segment, which functioned as the last mobile segment. This resulted in progressive failure culminating in spontaneous dislocation. Clinicians should recognize that, even without trauma, the last mobile segment in DISH may fail catastrophically. In such cases, rigid circumferential stabilization with long-segment fixation should be considered.

## Introduction

Diffuse idiopathic skeletal hyperostosis (DISH) is characterized by flowing ossification along the anterolateral aspect of the spine and has distinct radiographic and pathological features [1]. DISH is increasingly recognized as a clinically relevant whole-spine disorder associated with metabolic factors such as obesity, diabetes mellitus, and insulin resistance [2-4]. Recent data further suggest that DISH may be unexpectedly prevalent even in younger patients when severe obesity is present [4]. As ankylosis progresses, the spine loses segmental mobility and behaves biomechanically as a long lever arm, predisposing it to unstable lesions [5,6].

Although traumatic spinal lesions in ankylosed spines are well described, spontaneous segmental failure without a clear traumatic episode is uncommon. Several reports have described symptomatic pseudoarthrosis or instability at the remaining mobile segment in patients with DISH [7-11]. However, spontaneous lumbar dislocation with severe neurological deficit appears to be extremely rare. Herein, we present a case of spontaneous L4/L5 dislocation associated with DISH in a young patient with severe obesity and discuss its pathomechanism and surgical management.

## Case presentation

A 40-year-old man developed worsening low back pain approximately five months before transfer to our institution and visited a local hospital. No definite fracture was identified at that time, and he was discharged. His low back pain progressively worsened, and bilateral lower extremity weakness gradually developed. Because of his inability to ambulate, he remained at home, crawling for daily activities.

One month before transfer, he was emergently admitted to another hospital because of impaired consciousness and severe weakness. He was diagnosed with septic shock caused by a urinary tract infection and treated with antibiotics and intensive supportive care. Although the infection and altered mental status improved, persistent severe low back pain and neurological deficits remained. He was therefore referred to our department and transferred to our institution.

On admission to our institution, his height was 168 cm, and his weight was 132 kg, corresponding to a body mass index of 46.7 kg/m^2^. Other than severe obesity, the patient had no remarkable underlying comorbidities. The white blood cell count was 4.60 ×10^3/μL (reference range: 4.5-11.0 ×10^3/μL) and the C-reactive protein level was 2.47 mg/dL (reference range: <0.3 mg/dL). Neurological examination revealed marked bilateral lower extremity weakness: iliopsoas, 3+/2-; quadriceps, 3+/2; tibialis anterior, 1/1; extensor hallucis longus, 1/1; gastrocnemius, 1/1; and flexor hallucis longus, 1/1 using the Medical Research Council (MRC) scale for manual muscle testing. Sensory disturbance was present below the L1 dermatome bilaterally. Bladder-bowel dysfunction was also noted. His Japanese Orthopaedic Association (JOA) score was -3.

**Figure 1:**
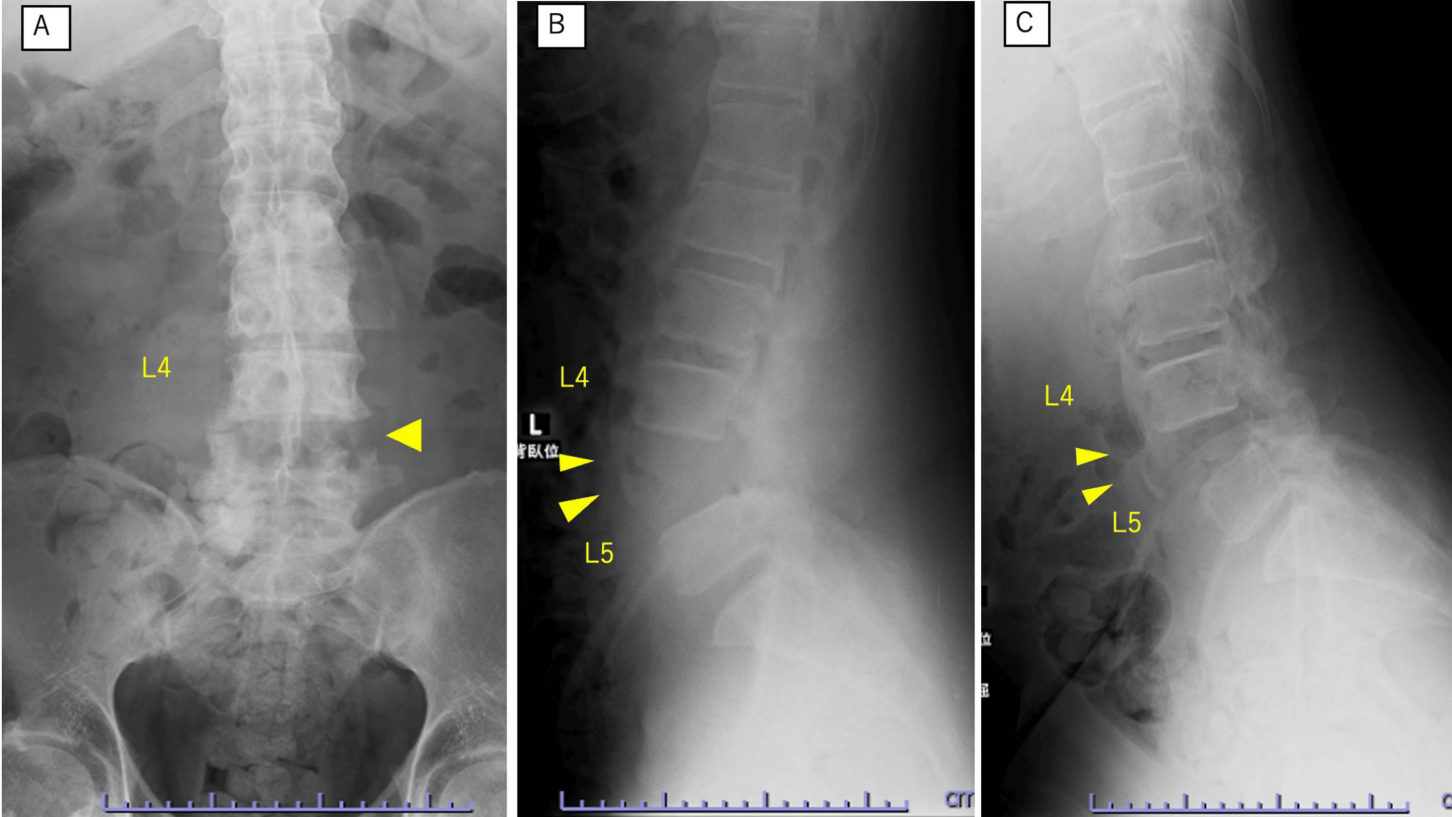
Preoperative dynamic radiographs demonstrating dislocation and instability at L4/L5. (A) Anteroposterior radiograph showing widening of the L4/L5 disc space (arrowheads). (B) Lateral radiograph obtained in the supine position showing widening of the L4/L5 disc space (arrowheads). (C) Lateral radiograph obtained in the lateral decubitus flexion position showing partial reduction of the disc space widening at L4/L5, indicating positional instability at the only remaining mobile segment (arrowheads).

Dynamic radiographs demonstrated positional instability at L4/L5, with widening of the disc space in the supine position and partial reduction in the lateral decubitus flexion position (Figure 1).

**Figure 2:**
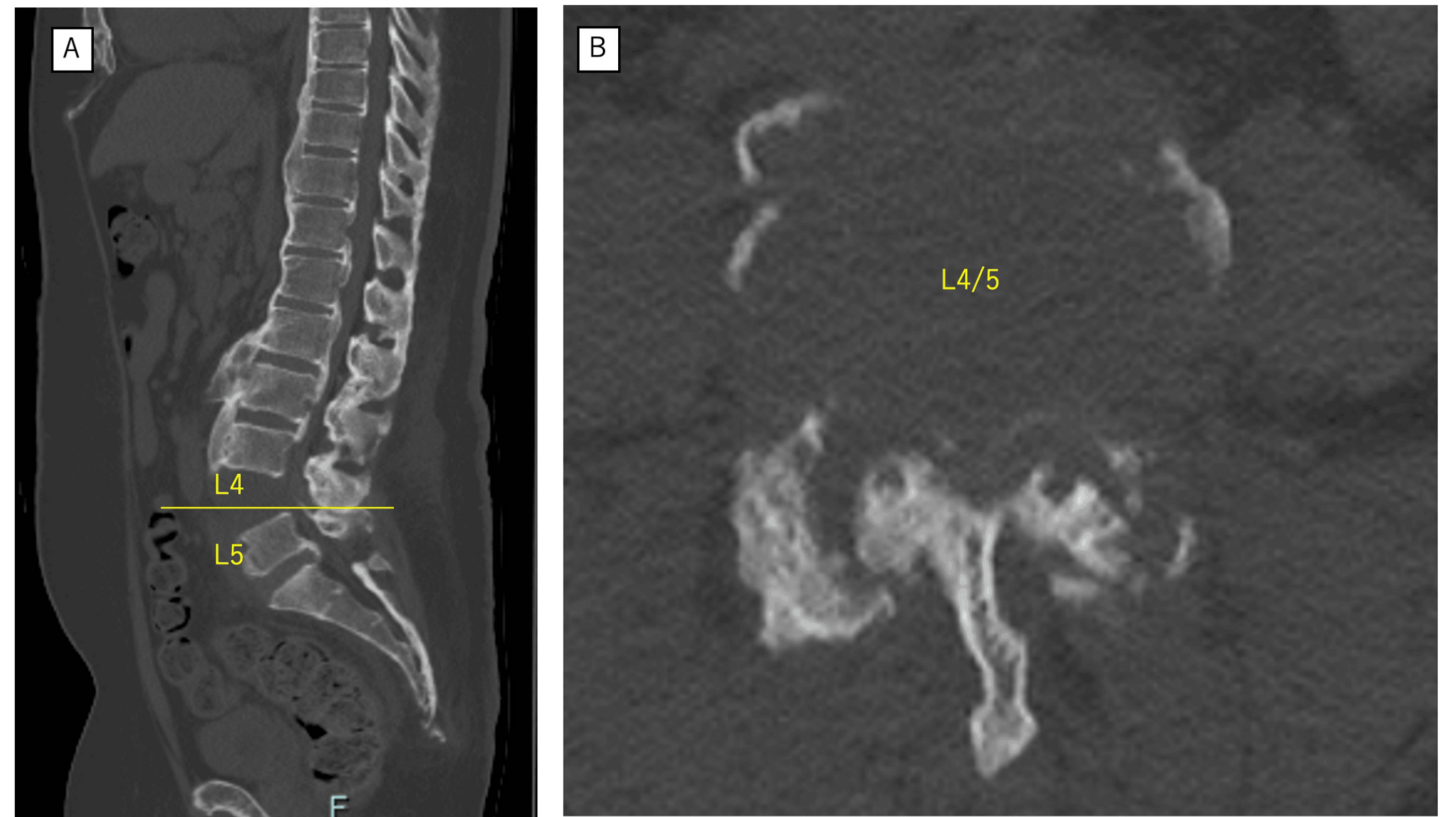
Preoperative computed tomography showing structural failure at L4/L5. (A) Sagittal reconstructed computed tomography image demonstrating extensive thoracolumbar ankylosis with spontaneous L4/L5 dislocation at the last mobile segment. (B) Axial computed tomography image at L4/L5 showing disruption of the posterior elements with facet separation.

Computed tomography revealed extensive osseous bridging throughout the thoracolumbar spine, consistent with DISH, with near-complete ankylosis of all lumbar segments except L4/L5. At L4/L5, spontaneous dislocation and facet joint separation were observed (Figure 2).

**Figure 3:**
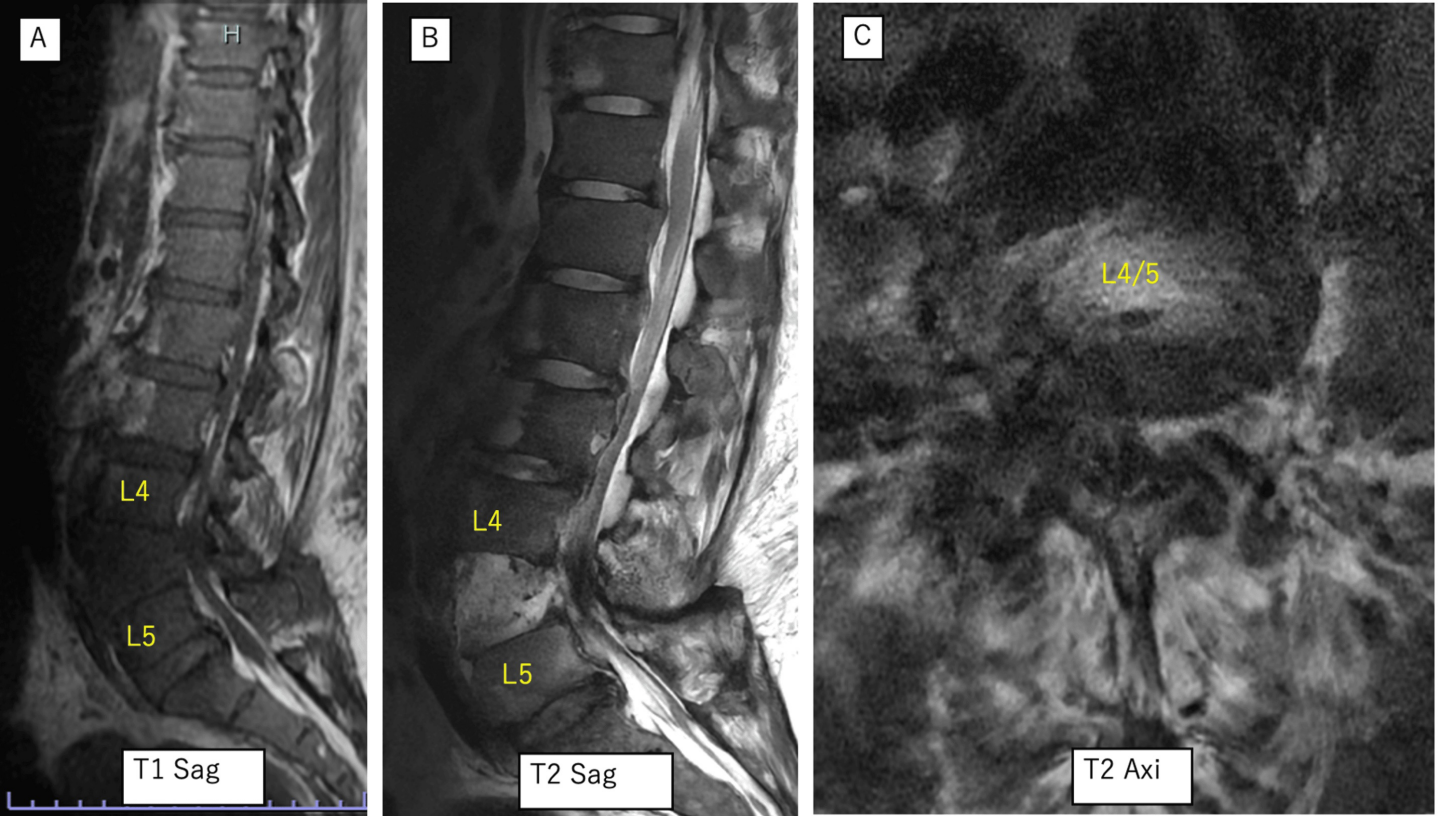
Preoperative magnetic resonance imaging demonstrating cauda equina compression at L4/L5. (A) Sagittal T1-weighted image; (B) sagittal T2-weighted image; (C) axial T2-weighted image showing severe compression of the cauda equina at the L4/L5 level.

Magnetic resonance imaging demonstrated compression of the cauda equina at L4/L5 (Figure 3).

Based on these findings, we diagnosed a spontaneous L4/L5 dislocation associated with DISH and cauda equina syndrome. Because the lesion was considered equivalent to a three-column injury with marked instability at the only remaining mobile segment, we planned staged circumferential reconstruction with combined posterior fixation and anterior column reconstruction.

First, percutaneous posterior fixation from L2 to S1 was performed in the lateral decubitus position using a four-rod construct. This approach was selected to avoid worsening the L4/L5 dislocation, which we feared might occur if the patient were placed prone before provisional stabilization. Second, after posterior stabilization had been achieved, the patient was repositioned prone, and S2 alar-iliac screws were inserted under navigation guidance. These were connected from L4 to S2, creating a six-rod construct and extending fixation to achieve a “three-above, three-below” strategy across the unstable segment. This second stage was necessary because S2 alar-iliac screw placement was technically difficult in the lateral decubitus position. Finally, in a semilateral position, anterior lumbar interbody fusion at L4/L5 was performed with cage insertion and iliac bone grafting to reconstruct anterior column support.

**Figure 4:**
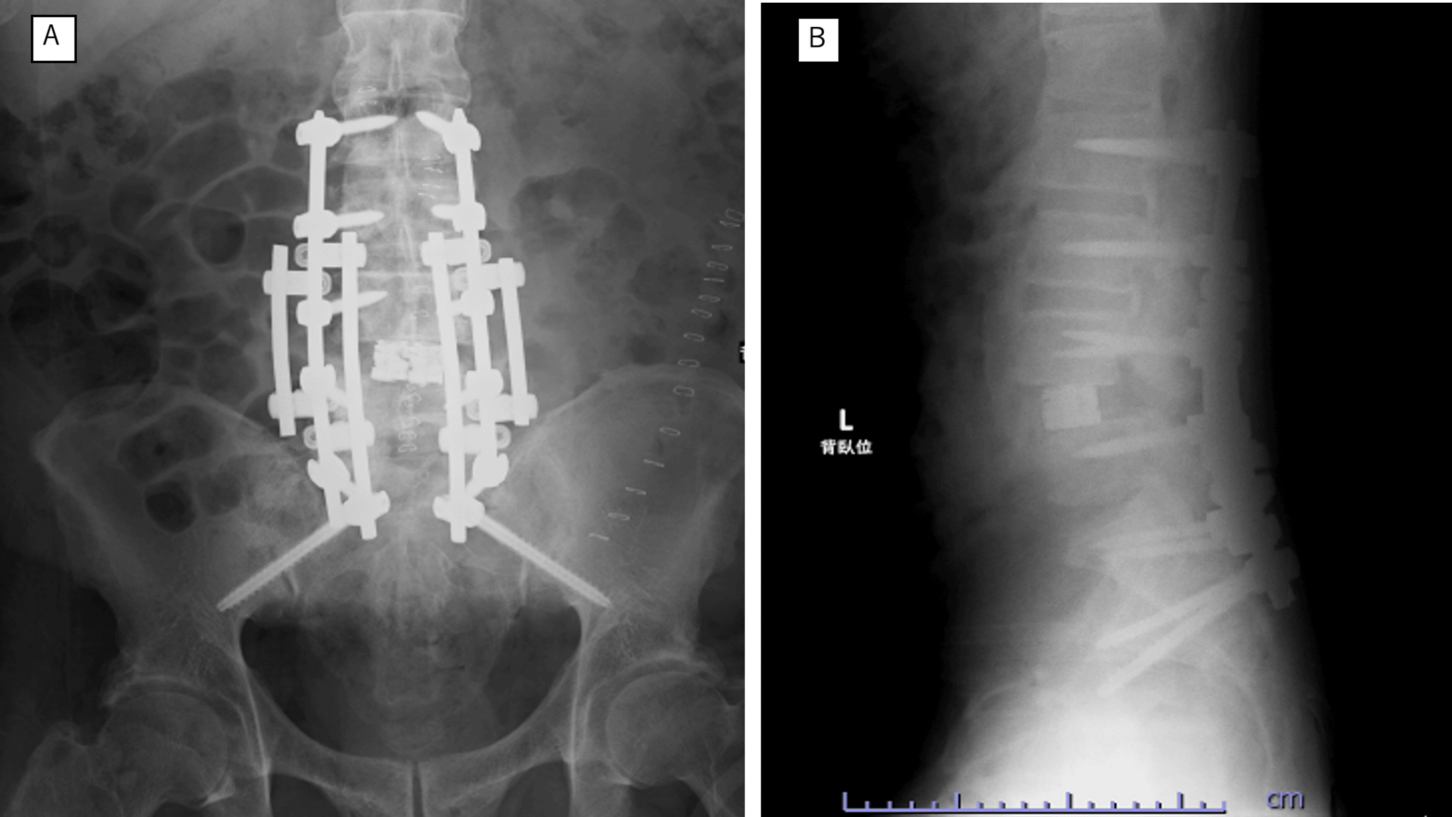
Postoperative radiographs after staged reconstruction. (A) Anteroposterior radiograph. (B) Lateral radiograph. Long-segment posterior fixation with pelvic anchorage and anterior reconstruction at L4/L5 achieved restoration of alignment and stabilization of the unstable segment.

At two months postoperatively, low back pain improved from a numeric rating scale score of 10 to 3. Iliopsoas and quadriceps strength showed mild recovery, but distal motor function below the tibialis anterior remained 1/1 bilaterally using the MRC scale for manual muscle testing. Sensation improved in the thighs and posterior legs, whereas numbness persisted over the dorsum of the feet, toes, buttocks, and perianal area. Bladder-bowel dysfunction remained, requiring intermittent self-catheterization with decreased defecation sensation and fecal incontinence. At six months postoperatively, at the final follow-up, the patient required a wheelchair for mobility. Radiographs confirmed maintained fixation and local stability after staged circumferential reconstruction (Figure 4).

## Discussion

This case highlights three important points. First, spontaneous catastrophic failure can occur at the last mobile segment in DISH even in the absence of a clear traumatic event. Second, severe obesity may amplify the chronic mechanical stress concentrated at that segment. Third, when such lesions are accompanied by gross instability and neurological compromise, rigid circumferential reconstruction with long-segment fixation may be required.

DISH is generally recognized as a disorder of older adults, but metabolic factors are believed to contribute to its development [2-4]. In addition to its classical radiographic definition, DISH is now recognized as a clinically relevant whole-spine disorder associated with obesity, diabetes mellitus, and insulin resistance [1-4]. Our patient was relatively young at 40 years of age, but he had severe obesity with a body mass index of 46.7 kg/m^2^. This metabolic background may have contributed both to the early development of DISH and to the excessive mechanical loading of the lumbar spine. Recent data have further suggested that DISH may be unexpectedly prevalent even in younger patients when severe obesity is present [4].

In ankylosed spines, the fused segments act as a long lever arm. As a result, stress tends to concentrate at transition zones and residual mobile levels [5,6]. This biomechanical vulnerability has been well recognized in unstable lesions of ankylosed spines, including fractures and pseudoarthrosis [5,6]. Several reports have described spontaneous symptomatic pseudoarthrosis in patients with DISH. Quagliano et al. reported vertebral pseudoarthrosis associated with DISH [7]. Miyamoto et al. reported symptomatic pseudoarthrosis at T11/T12 [8], and Hasegawa et al. described a similar lesion at L2/L3 [9]. Funayama et al. reported pseudoarthrosis with dynamic instability at L4/L5, which had become the only mobile segment in a patient with extensive DISH [10]. Chi et al. also reported a symptomatic mobile lumbar segment with canal stenosis at the caudal end of a fused spine associated with DISH [11]. Taken together, these reports support the concept that the remaining mobile segment in DISH may progressively fail under chronic stress.

Our case appears to represent a more advanced and destructive form of this process. Nearly all lumbar segments except L4/L5 were ankylosed, meaning that L4/L5 functioned as the last mobile segment and hinge point. In addition, L4/L5 is inherently one of the more mobile lumbar levels. Severe obesity likely imposed additional axial and shear loads across the segment. We speculate that the combination of (1) being the only residual mobile segment, (2) being a highly mobile lumbar level, and (3) carrying excessive load due to severe obesity led to chronic stress concentration. Over time, this may have caused failure of the posterior elements, including superior articular process fracture and facet separation, ultimately progressing to spontaneous dislocation. In this respect, the present case can be interpreted as the extreme end of the spectrum of the last mobile segment failure in DISH.

Most previously reported spontaneous lesions in DISH have presented primarily with pain or progressive instability rather than profound neurological deficit [7-11]. In contrast, our patient developed severe bilateral lower extremity weakness and bladder-bowel dysfunction due to cauda equina compression. To our knowledge, spontaneous L4/L5 dislocation associated with DISH and presenting with such severe neurological compromise is extremely rare. The present case, therefore, expands the clinical spectrum of spontaneous unstable lesions in DISH and suggests that failure of the residual mobile segment may culminate not only in pseudoarthrosis or dynamic instability but also in frank dislocation with marked neurological deterioration.

The surgical strategy in this case was based on the concept that this lesion should be treated not as an ordinary degenerative lumbar disorder but as a highly unstable injury in an ankylosed spine. Previous studies of spinal fractures and unstable lesions in ankylosed spines have emphasized the need for long-segment rigid fixation because of the long lever arms involved [5,6]. Okada et al. also demonstrated that fractures in patients with DISH show characteristic clinical features depending on the spinal level, further supporting the importance of understanding the unique biomechanics of DISH-associated lesions [6]. Hasegawa et al. reported failure after short fixation in a spontaneous pseudoarthrosis case, underscoring the importance of adequate fixation length [9]. In our patient, operative positioning itself was a major concern because the L4/L5 dislocation was considered likely to worsen in the prone position before stabilization. For this reason, the first stage consisted of posterior fixation from L2 to S1 in the lateral decubitus position to obtain provisional stability. Pelvic fixation with S2 alar-iliac screws was then added in a second stage after repositioning the patient prone, because screw insertion was technically difficult in the lateral position. We then completed anterior column reconstruction at L4/L5. Thus, staged circumferential reconstruction in this case was selected not only for biomechanical reasons but also to safely manage the positional risk of aggravating the dislocation. Although neurological recovery was limited, pain relief and local stability were successfully achieved.

This report has several limitations. It describes a single case with a relatively short follow-up. Moreover, because the patient had a prolonged preoperative course with sepsis, poor general condition, and delayed transfer, the extent to which earlier intervention might have improved neurological recovery is uncertain. In addition, although this case strongly suggests catastrophic failure of the last mobile segment in DISH, the precise temporal sequence of instability progression cannot be fully reconstructed. Nevertheless, the present case provides an important clinical warning that the last mobile segment in DISH may fail spontaneously and catastrophically, particularly in the presence of severe obesity and marked mechanical stress concentration.

## Conclusions

We report a rare case of spontaneous L4/L5 dislocation associated with DISH in a young patient with severe obesity. In this case, L4/L5 functioned as the last mobile segment within an otherwise ankylosed thoracolumbar spine, likely resulting in chronic stress concentration and segmental failure. Even in the absence of trauma, clinicians should be aware that the remaining mobile segment in DISH can become a site of catastrophic instability. When marked instability is accompanied by neurological compromise, long-segment circumferential stabilization should be considered.
